# Treatment of high-grade glioma patients during the COVID-19 pandemic: Impact on overall survival, tumor size and delay of treatment

**DOI:** 10.1371/journal.pone.0287993

**Published:** 2023-06-30

**Authors:** Mario Mischkulnig, Benjamin Hopp, Lisa I. Wadiura, Farjad Khalaveh, Barbara Kiesel, Karl Rössler, Georg Widhalm, Christian Dorfer

**Affiliations:** Department of Neurosurgery, Medical University Vienna, Vienna, Austria; Goethe University Hospital Frankfurt, GERMANY

## Abstract

**Background:**

Throughout the last years, the coronavirus disease 2019 (COVID-19) pandemic posed a major challenge to the optimal and timely treatment of neurooncological patients around the world. While the importance of prompt surgical treatment in high-grade gliomas is widely accepted, there is sparse data on the impact of the pandemic on patients suffering from this malignant disease.

**Methods:**

We performed a retrospective analysis of patients undergoing surgical high-grade glioma treatment at the Medical University of Vienna between March 2020 and February 2021, as well as a control cohort of patients who received treatment between January and December 2019. Time lag between referral for surgical treatment to actual surgery, preoperative tumor volume and overall patient survival were compared between groups.

**Results:**

A total of 118 patients, including 62 cases treated during the first year of the COVID-19 pandemic, as well as 56 control patients, were investigated in this study. Median interval to surgery was significantly shorter in patients treated during COVID-19 compared with the control group (4.00 versus 7.00 days; p = 0.0005). In contrast, patients treated during COVID-19 exhibited marginally larger preoperative tumor volumes, while overall patient survival was comparable between groups.

**Conclusions:**

The COVID-19 pandemic did not negatively affect the overall survival of patients undergoing surgical high-grade glioma treatment at our institution. The significantly shorter treatment delay in patients treated during the pandemic likely reflects increased resource allocation for this critical patient population.

## Introduction

High-grade gliomas (HGG) constitute the most common malignant primary central nervous system tumors and are associated with poor prognosis and significant mortality (typical median overall survival time: < 2 years) [1, 2]. Besides adjuvant concomitant chemo- and radiotherapy treatment, the timely surgical intervention by means of maximal safe resection or biopsy for histopathological tumor analyses in cases not amenable to safe surgical excision constitutes the most crucial treatment for HGG patients [3]. Throughout the last years, the far-reaching impact of the coronavirus disease 2019 (COVID-19) pandemic on health care systems around the world has threatened optimal patient care and, thus, may have caused treatment delays even in cases of critical, life-threatening diseases, such as brain tumors [4–6].

Given the importance of timely diagnosis and treatment in neurooncological patients, there was an early consensus that all possible measures should be taken to prevent treatment delay in these patients [3]. According to a prospective study performed in the United Kingdom between April 1^st^ and May 31^st^ 2020, however, changes in HGG management as compared to usual care before COVID-19 were observed in 9% of primary cases as well as 17% of recurrent cases [7]. Considering the potential severe consequence that any treatment delay may have in HGG patients, investigation of the impact of the COVID-19 pandemic on these patients is of paramount importance.

A previous study assessed tumor volumes at diagnosis in a case series of patients who suffered from different brain tumor entities and received surgery in Wuhan, China, between April 2020 and May 2020, and reported significantly larger preoperative tumor volumes in these patients compared with patients treated one year earlier [8]. A case series study conducted in Dublin, Ireland, between March 2020 and May 2020 investigated the effects of the COVID-19 pandemic on the timing of treatment in a series of 60 patients, revealed a shorter mean interval between hospital admission and surgery in patients during the pandemic than in patients treated before the pandemic (2.3 versus 2.8 days) [9]. Moreover, the investigators reported slightly improved immediate postoperative (30-day) outcome rates during the pandemic [9]. Although another study in a case series of 23 HGG patients who were treated in March and April 2020 also suggested uncompromised treatment and safety of patients, a detailed analysis of long-term patient survival has not yet been provided [10].

The aim of this study was to provide a comprehensive analysis of the impact of the COVID-19 pandemic on HGG patient survival in relation to tumor volume at time of surgery and potential treatment delay after initial presentation, as compared with a historic control cohort of HGG patients who were treated before the pandemic.

## Material and methods

We performed a retrospective single-center study in patients undergoing initial surgical treatment of a HGG at the Department of Neurosurgery, Medical University of Vienna. Patients treated during the first year of the pandemic were compared with a control cohort of patients treated before the pandemic. Overall patient survival, preoperative volumetric tumor size and interval between the time of initial referral for surgery and date of surgery were analyzed. This study was approved by the local ethics committee at the Medical University of Vienna (EK 1231/2022).

### Patient selection

For the COVID-19 patient cohort, all patients older than 18 years of age at the time of surgery and undergoing surgical treatment (either resection or biopsy) of a newly diagnosed, histopathologically verified high-grade glioma (i.e., World Health Organization [WHO] grade 3/4) between March 1^st^ 2020 and February 28^th^ 2021 at the Department of Neurosurgery, Medical University of Vienna, were included in this investigation. The control cohort consisted of patients fulfilling the same criteria as patients in the COVID-19 cohort and who received surgical HGG treatment in the year before the pandemic (between January 1^st^ 2019 and December 31^st^ 2019). Patients treated in January and February 2020 were excluded from both the COVID-19 and the control cohort as no formal restrictions were active in Austria during this period despite increasing media coverage and public awareness which may have already influenced patient behavior. Patients with previously treated brain tumors and cases with no preoperative Magnetic Resonance Imaging (MRI) images available for analysis were also excluded. Data collection was performed between April 16^th^ and December 12^th^ 2022, the authors had access to information that could identify individual participants during data collection. The inclusion and exclusion criteria for this study are shown in Table 1.

**Table 1:** Inclusion and exclusion criteria for cases and controls.

**Inclusion criteria for cases** *
1. Male or female patients, ≥ 18 years of age at time of resection, ≤ 99 years of age at time of surgery
2. Patients with histologically confirmed high-grade gliomas WHO^†^ grade 3/4
3. Initial resection/biopsy at the Department of Neurosurgery at the Medical University of Vienna between March 1^st^ 2020 and February 28^th^ 2021
**Exclusion criteria for cases** *
1. Male or female patients, < 18 years of age at time of resection, > 99 years of age at time of surgery
2. Patients with unclear histology or histology other than high-grade gliomas WHO grade 3/4 or brain metastases
3. No preoperative contrast-enhanced T1 MRI^‡^ images available
4. Prior brain tumor treatment
**Inclusion criteria for controls** **
1. Male or female patients, ≥ 18 years of age at time of resection, ≤ 99 years of age at time of surgery
2. Patients with histologically confirmed WHO high-grade gliomas WHO grade 3/4
3. Initial resection/biopsy at the Department of Neurosurgery at the Medical University of Vienna performed between January 1^st^ 2019 and December 31^st^ 2019
**Exclusion criteria for controls** **
1. Male or female patients, < 18 years of age at time of resection, > 99 years of age at time of surgery
2. Patients with unclear histology or histology other than high-grade gliomas WHO grade 3/4 or brain metastases
3. No preoperative contrast-enhanced T1 MRI images available
4. Prior brain tumor treatment

* Patients surgically treated during the first year of the COVID-19 pandemic

** Patients who received surgical treatment before the COVID-19 pandemic

^†^ World health organization

^‡^ Magnetic resonance Imaging

### Data collection

**Fig 1:**
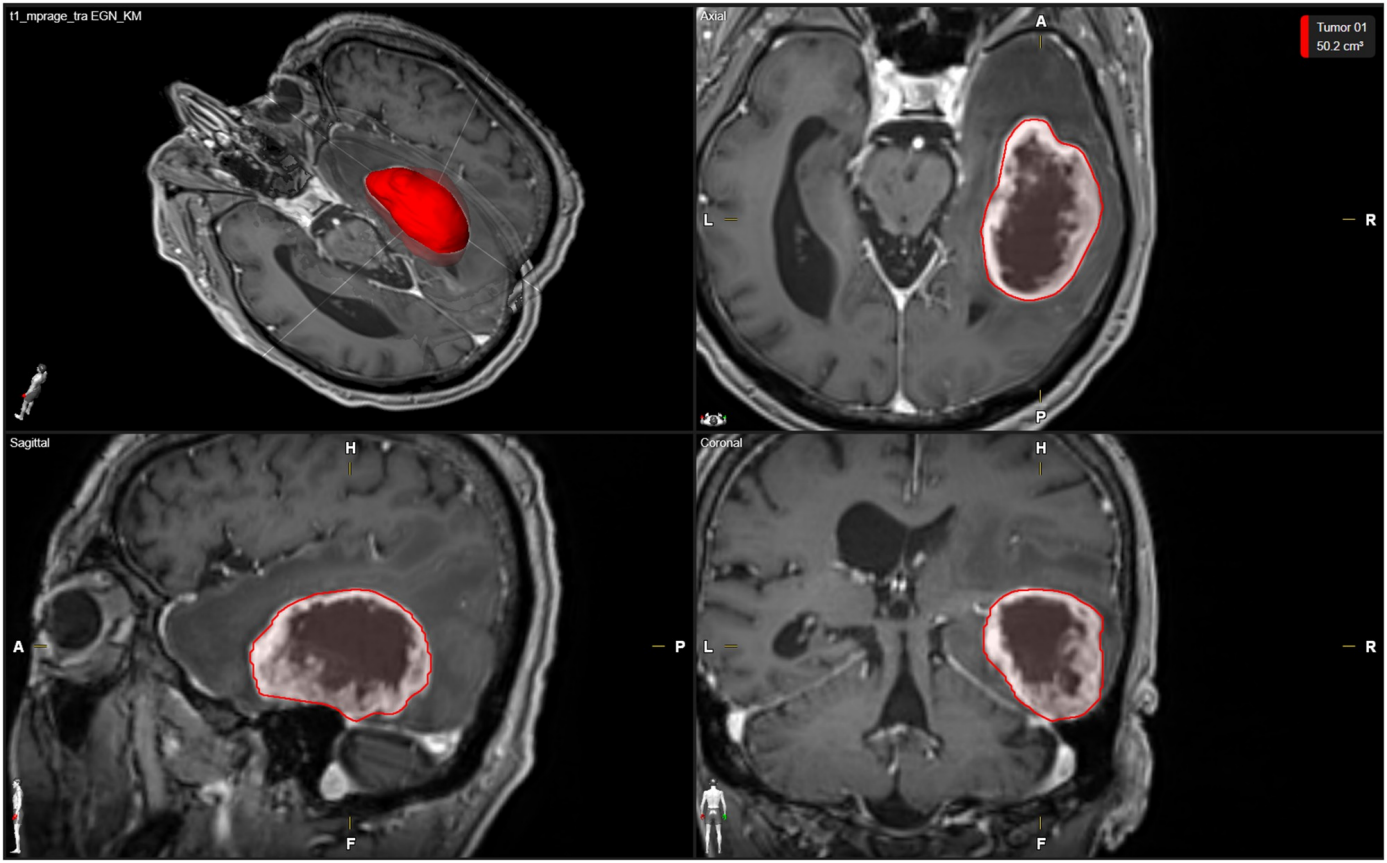
Example of volumetric analysis. The volumetric analysis of a right temporal HGG is shown in a 3-dimensional reconstruction, as well as in axial, sagittal and coronal planes. The tumor volume as determined based on contrast-enhanced T1-weighted images is highlighted in red.

Tumor volumetry was performed based on the last preoperative contrast-enhanced T1 weighted images using the Brainlab neurosurgical navigation software (Brainlab AG, Munich, Germany). The measured tumor volumes included areas of significant contrast enhancement and the necrotic tumor core. Areas with non-specific contrast-enhancement or only T2 signal alterations were not included in the volumetric analysis. A typical volumetric analysis of preoperative tumor volume using the Brainlab neurosurgical navigation software is shown in Fig 1.

Overall survival data were retrieved using the mortality data comparison service of the IT Systems & Communications (ITSC) department of the Medical University of Vienna, which provides mortality data from Statistics Austria for medical research and quality control. Information on the last follow up was retrieved using the patient administration database held by the Medical University of Vienna and survival was examined on September 9^th^ 2022.

The time lag between referral for surgery to surgery was defined as the time interval between the date of the surgical consult at our department when the need for surgical treatment was first indicated and the date when surgical treatment was performed. The time from surgery to adjuvant treatment was defined as the time interval between the date of the initial surgical treatment and the date when adjuvant treatment was initiated.

In order to detect pre- and perioperative cases of COVID-19 infections in the patients treated during the pandemic, all SARS-CoV2 tests performed during admission at our department were reviewed and patient documents including outpatient reports, surgical reports and discharge letters were screened for mention of any infections interfering with tumor diagnosis or neurosurgical treatment.

### Statistical analyses

Statistical analyses were performed using the proprietary software package SPSS Statistics Version 27.0 (IBM, Armonk, NY, USA). For descriptive characterization of the entire patient cohort, as well as the two study groups (COVID-19 versus pre-COVID-19 control cohorts), nominal/ordinal variables such as gender, WHO tumor grade, isocitrate dehydrogenase (IDH) mutation status, O6-Methylguanin-DNA-Methyltransferase (MGMT) promoter methylation status, type of surgery (complete resection / incomplete resection / biopsy), type of adjuvant treatment were presented as counts and percentages. For metric variables, including patients age, tumor volumes, time to surgery available beds at our neurosurgical ICU and time from surgery to adjuvant treatment, the mean and standard deviation in case of normally distributed data, or median and interquartile range (IQR) in case of non-normally distributed data were reported. To rule out relevant differences in patient characteristics between the COVID-19 and control groups, differences in metric and nominal variables were analyzed employing parametric/non-parametric tests (as described below) and chi^2^ tests, respectively. Inferential statistical analysis for differences in the metric tumor volume and interval to surgery variables was performed using an initial testing for normal distribution according to the Shapiro-Wilk test. In case of normal distribution, the subsequent test for differences between the COVID-19 and control groups was performed using the unpaired student’s t-tests, whereas in case of absent normal distribution, the non-parametric Mann-Whitney-U test was applied. For survival analysis, Kaplan-Meier curves with log-rank test for group differences were used.

## Results

### Patient selection

A total of 544 adult patients treated for suspected brain tumors during the first year of the COVID-19 pandemic were identified. Histopathological diagnosis of high-grade glioma was verified in 97 cases and 62 of these patients had not received previous brain tumor treatments. In contrast, 593 adult patients treated for suspected brain tumors during the control period were identified. High-grade glioma diagnosis was established in 85 patients, of whom 56 had not received previous brain tumor treatments. Preoperative contrast-enhanced T1 MRI images were available in all patients. Therefore, a total of 118 patients were included in the study with 62 patients (52.5%) treated during the first year of the COVID-19 pandemic (cases) and 56 patients (47.5%) undergoing surgery prior to the pandemic (controls).

### Patient characteristics

The median age in the entire cohort was 62 years (range: 26–88 years) and did not differ significantly between cases and controls (63 years, range: 26–84 versus 61 years, range: 29–88). The male / female ratio was 1.36 and there was no significant difference in the gender distribution between cases and controls. In the entire cohort, a majority of 96 (81.4%) patients was diagnosed with WHO grade 4 gliomas, with the remaining 22 (18.6%) patients presenting WHO grade 3 gliomas. While the proportion of WHO grade 4 gliomas was slightly lower in the COVID-19 group (52 of 62 cases; 83.9%) than in the control group (44 of 56 cases; 78.6%), there was no statistically significant difference between the two study groups. A majority of 47 patients (83.9) in the COVID-19 group and 53 patients (85.5%) in the control group was diagnosed with IDH wildtype tumors, while IDH mutations were detected in 9 patients in each group. IDH mutation status did not significantly differ between the two study groups. MGMT promoter methylation was present in a slightly lower percentage of cases in the COVID-19 group (21 of 62 cases, 33.9%) compared to the control group with 27 of 56 cases (48.2%) but there was no significant difference between both groups. Complete resection was performed in a majority of cases in both the COVID-19 group (43.5%, 27 of 62 cases) and control group (35.7%, 20 of 56 cases) and there was no significant difference in regard to type of surgery between both groups. The number of available beds at our neurosurgical ICU in the COVID-19 cohort as well as the control group and the entire study population was 9.00 beds (8.00–9.00). Combined radiochemotherapy was the most common adjuvant treatment in patients of the COVID-19 group (77.4%) as well as control group (64.2%). The median time interval from surgery to adjuvant treatment was shorter in the patients treated during the COVID-19 pandemic with 28 days (22.50–31.00) compared to 34 days (26.00–42.00). No preoperative COVID-19 infections resulting in delayed diagnosis or neurosurgical treatment were detected and no perioperative infections until discharge from our department occurred in the patient cohort treated during the pandemic. A detailed overview of patient characteristics of the comparison between the two study groups is provided in Table 2.

**Table 2:** Patient characteristics.

		Overall		COVID-19 Group		Control Group		*p-value*
		*n*	(%)	*n*	(%)	*n*	(%)	
**Number of Patients**		**118**	**(100.0)**	62	(52.5)	56	(47.5)	
**Age in years**	**Median (range)**	**62 (26–88)**		63 (26–84)		61 (29–88)		
**Gender**								
	**Male**	**68**	**(57.6)**	33	(28.0)	35	(29.7)	
	**Female**	**50**	**(42.4)**	29	(24.5)	21	(17.8)	
WHO* **Grade**								
	**WHO Grade 3**	**22**	**(18.6)**	10	(8.5)	12	(10.2)	
	**WHO Grade 4**	**96**	**(81.4)**	52	(44.1)	44	(37.2)	
**IDH mutation status**								
	**IDH wildtype**	**100**	**(84.7)**	53	(85.5)	47	(83.9)	
	**IDH mutated**	**18**	**(15.3)**	9	(14.5)	9	(16.1)	
**MGMT status**								
	**Methylated**	**48**	**(40.7)**	21	(33.9)	27	(48.2)	
	**Unmethylated**	**66**	**(55.9)**	38	(61.3)	28	(50.0)	
	**Missing data**	**4**	**(3.4)**	3	(4.8)	1	(1.8)	
**Type of surgery**								
	**Complete resection**	**47**	**(39.8)**	27	(43.5)	20	(35.7)	
	**Incomplete Resection**	**41**	**(34.7)**	22	(35.5)	19	(33.9)	
	**Biopsy**	**30**	**(25.5)**	13	(21.0)	17	(30.4)	
**Available beds at neurosurgical ICU**								
	**Median (IQR)**	**9.00 (8.00–9.00)**		9.00 (8.00–9.00)		9.00 (8.00–9.00)		
**Interval surgery to adjuvant treatment**								
	**Median (IQR)**	**29.00 (24.00–36.00)**		28 (22.50–31.00)		34 (26.00–42.00)		
**Type of adjuvant treatment**								
	**Radiochemotherapy**	**84**	**(71.2)**	48	(77.4)	36	(64.2)	
	**Radiotherapy only**	**4**	**(3.4)**	1	(1.6)	3	(5.4)	
	**Chemotherapy only**	**3**	**(2.5)**	0	(0.0)	3	(5.4)	
	**No oncological treatment**	**15**	**(12.7)**	9	(14.5)	6	(10.8)	
	**Missing data**	**12**	**(10.2)**	4	(6.5)	8	(14,2)	
**Preoperative tumor volume in cm^3^**								
	**Median (IQR** ** **)**	**16.85 (2.73–36.40)**		20.95 (2.61–41.20)		13.00 (3.32–30.2)		*0*.*500*
**Interval to surgery in days**								
	**Median (IQR)**	**5.00 (3.00–11.00)**		4.00 (2.00–8.00)		7.00 (5.00–15.00)		*<0*.*0005*
**Patient survival**								
	**Median (95% CI** ^†^ **)**	**493 Days (345–641)**		493 Days (369–617)		401 Days (0–830)		*0*.*914*
	**Estimated 1-year survival rate**	**61.0%**		64.9%		56.5%		

* World Health Organisation

** Interquartile range

^†^ Confidence Interval

### Preoperative tumor volume

**Fig 2:**
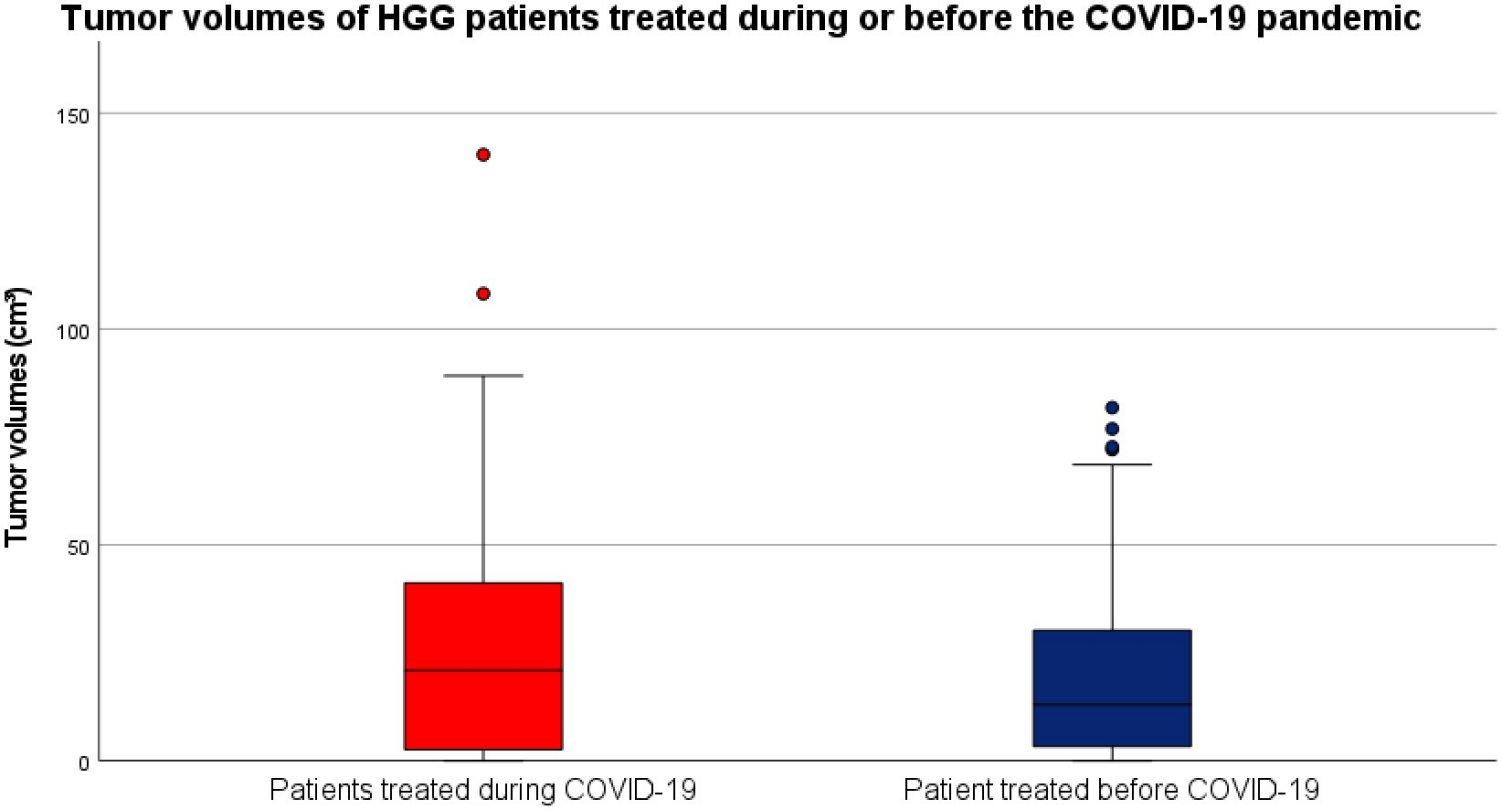
Tumor volumes of HGG patients during and before the COVID-19 pandemic. Boxplot diagrams of preoperative tumor volumes are shown for patients treated during the first year of the COVID-19 pandemic (red) or before COVID-19 (blue). While median volumes were slightly higher in patients treated during the first year of the COVID-19 pandemic, no statistically significant difference was detected between the two study groups.

Preoperative MRI images were obtained 2.50 days (1.00–5.00) prior to surgery and there was no statistically significant difference between cases and controls (2.00 days [1.00–4.00] versus 4.00 days[1.00–6.00]). The median overall tumor volume in the entire cohort of 118 patients was 16.85 (2.73–36.40) cm^3^. While the median tumor volumes observed in the patients treated during the first year of the COVID-19 pandemic was slightly larger than the ones observed in the control group (20.95 [2.61–41.20] versus 13.00 [3.32–30.2] cm^3^), the group comparison by means of the Mann-Whitney-U test did not detect a significant difference between the two study groups (p = 0.500). A boxplot diagram of the observed tumor volumes in HGG patients treated during and before the COVID-19 pandemic is shown in Fig 2.

### Interval to surgery

**Fig 3:**
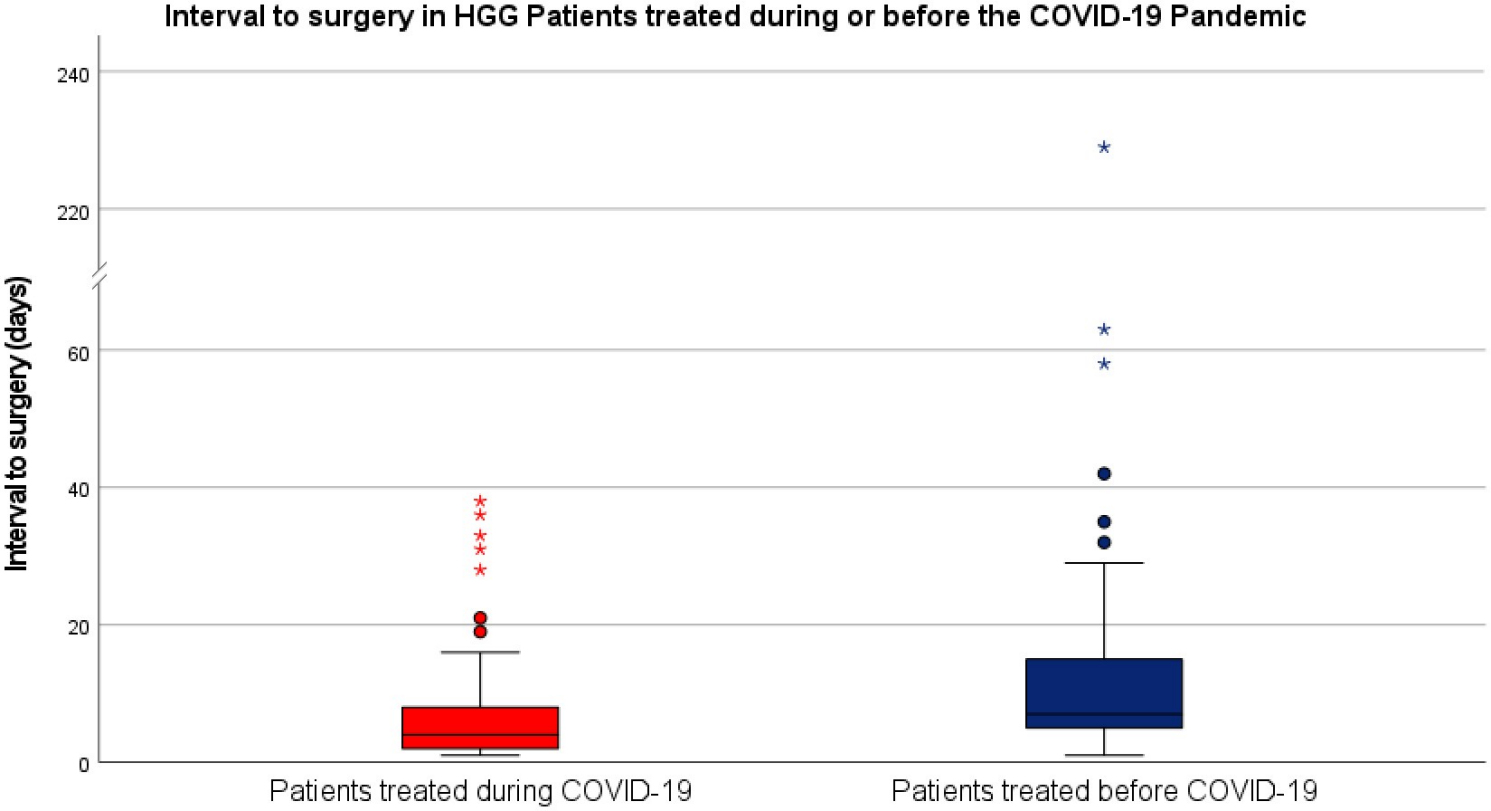
Interval to surgery in HGG patients during and before the COVID-19 pandemic. Boxplot diagrams of the interval to surgery are shown for patients treated during the first year of the COVID-19 pandemic (red) or before COVID-19 (blue). Median interval to surgery was significantly shorter in patients treated during the first year of the COVID-19 pandemic.

The overall median interval between initial referral for surgical treatment and the date of surgery was 5.00 (3.00–11.00) days in the entire cohort. This time lag was significantly shorter in patients treated during the first year of the pandemic than in the control cohort of patients treated before the COVID-19 pandemic (median interval to surgery: 4.00 [2.00–8.00] versus 7.00 (5.00–15.00) days; p < 0.0005). There was one extreme outlier among the treatment delay data points in the control cohort of patients treated before COVID-19, with a time interval of 229 days between initial referral for surgical treatment and surgery due to the patient initially refusing treatment. This patient was retained in the analysis as exclusion of such patients was not defined in the study protocol and a certain degree of delay due to patient preference, in general, cannot be ruled out retrospectively with certainty. We did, however, assess the effect of this outlier on the statistical analysis and found that inferential statistical testing of treatment the interval to surgery resulted in identical results upon exclusion of this patient. Boxplot diagrams of the observed interval to surgery in HGG patients treated during or before the COVID-19 pandemic are shown in Fig 3.

### Patient survival

**Fig 4:**
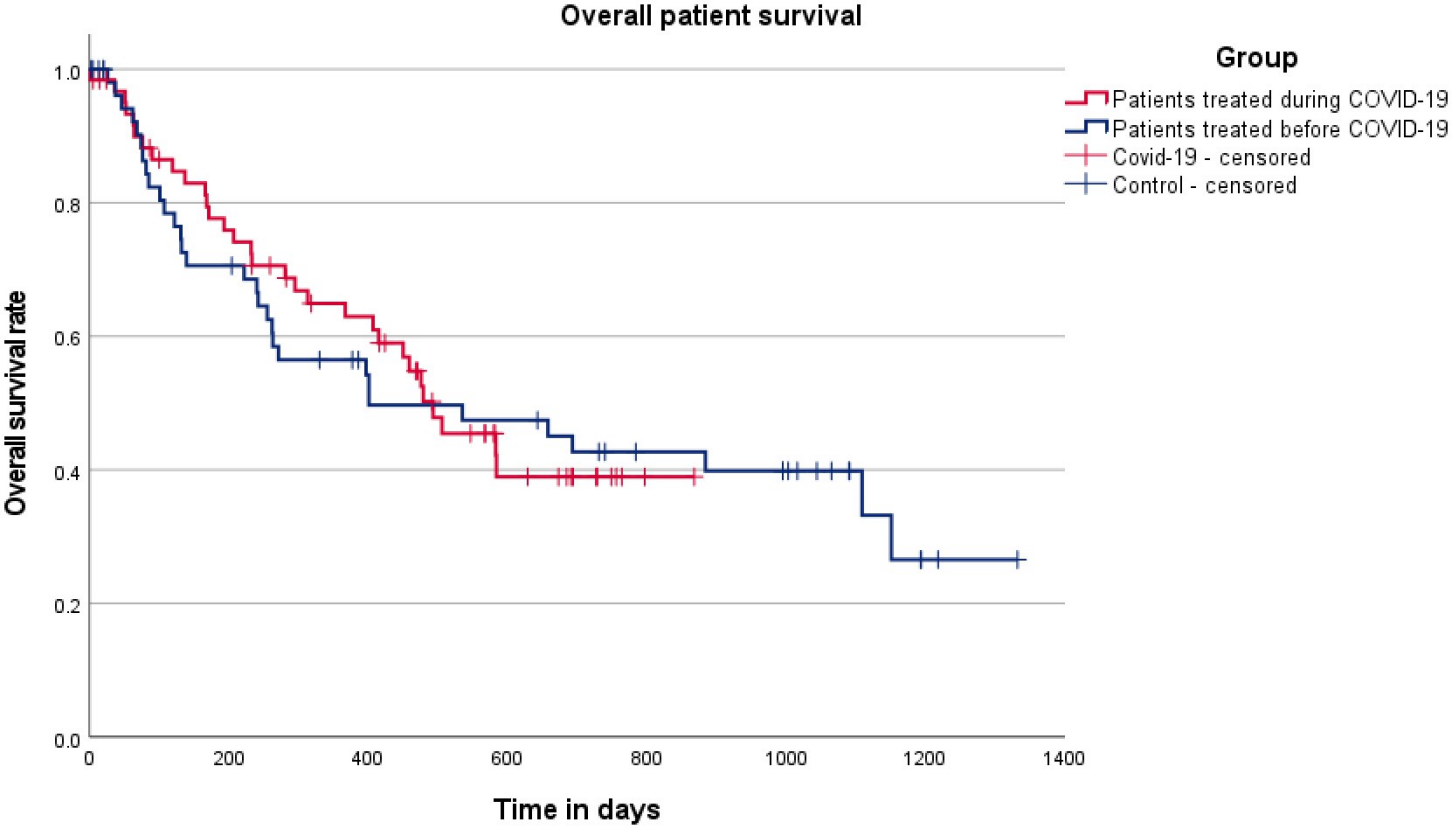
Overall patient survival in HGG patients during and before the COVID-19 pandemic. Kaplan-Meier curves of overall patient survival are shown for patients treated during the first year of the COVID-19 pandemic (red) or before COVID-19 (blue). There was no significant survival difference between patients treated during the first year of the COVID-19 pandemic and patients treated before COVID-19.

Overall, 62 patients (52.5%) succumbed to the disease while the remaining 56 patients (47.5%) were alive at the time of censoring. The proportion of confirmed deceased patients was slightly lower in the COVID-19 study group compared with the control group (50.0% versus 55.4%). Median patient survival in the entire cohort was 493 days (95% CI: 345–641 days), which was marginally higher in the patients treated during the first year of the COVID-19 pandemic compared with the control group (491 days [95% CI: 269–617 days] versus 401 days [95% CI: 0–830 days]). The estimated 1-year survival rate was 61.0% in the entire cohort and was slightly higher in the COVID-19 cohort (64.9%) compared to the control cohort (56.5%). Overall, the log-rank test did not detect any significant differences in patient survival between the two study groups (p = 0.914). Kaplan Meier curves of survival rates in patients treated during or before the COVID-19 pandemic are shown in Fig 4.

## Discussion

Maintenance of prompt and adequate treatment of brain tumor patients was early identified as a crucial goal for neurosurgeons confronted with the restrictions due to the COVID-19 outbreak [3]. While a commitment for the optimal treatment of patients suffering from malignant neurooncological diseases, such as HGG, was quickly reached, recommendations needed to consider the unprecedented and critical situation in many health care systems [11, 12]. Given the shortage of medical resources, such as reduced operating room and ICU capacities, and the reported rates of COVID-19-positive cases among some series of neurosurgical patients (> 10%), uncertainty prevailed as to whether the same quality of care and outcomes could be provided for all neurooncological patients [13]. While priority was clearly given to surgical HGG treatment, overall survival rates of HGG patients may have been impacted by many other factors, including delayed or waived concomitant treatments [3, 14].

Our analysis strongly suggests that there was no deterioration in the survival of HGG patients treated during compared to patients treated before the pandemic at our institution. Specifically for high-grade gliomas, one single-site study from Dublin, Ireland analyzed the 30-day postoperative morbidity and mortality rates in a cohort of 116 patients and found this to be lower during the pandemic [9]. These favorable findings in regard to overall mortality may, however, not be generalizable to centers world-wide since there is convincing evidence that 30-day mortality in neurooncological patients during the pandemic was significantly higher in low and lower middle income countries (OR 2,83) and upper middle income countries (OR 1.49) compared to high income countries [14]. As to potential reasons for this disparity in prognosis according to location by World Bank income groups, this large prospective study across 55 international centers in 26 countries demonstrated that the rates of any change to care (28% vs. 21%) and delayed surgery in particular (18% vs. 14%) was higher in low & low-middle income countries compared to high income countries [14]. A study from the United Kingdom as a high income country found that especially in newly diagnosed malignant brain tumors delivery of surgical treatment could mostly be maintained even during the first month of the pandemic while notable disruptions were primarily detected in adjuvant treatment and recurrent cases [7].

Interestingly, our analysis also revealed that the median interval from initial referral for surgical treatment to actual surgery at our center in Austria as a high income country was significantly shorter in patients treated during the COVID-19 pandemic. While this observation seems counterintuitive given the expected constraints on health care systems and the profound lack of resources due to the pandemic, this reduced interval from initial referral for surgery is consistent with findings reported by Ammon and colleagues [9]. It is also of note that the median time to surgery of 5 days observed in the entire study cohort is shorter than the one reported in a previous multicenter study with 13 days [15]. One major methodological difference that in part explains this disparity is that our study investigated the interval from initial consultation at our department to surgery rather than initial diagnostic MRI to surgery [15]. Furthermore, preoperative tumor board review is typically waived at our department in cases of radiologically suspected HGG whereas it was discussed as contributing factor to treatment delay by the authors of the previous investigation [15].

Also noteworthy were higher preoperative tumor volumes in patients treated during the COVID-19 pandemic, which corroborates the results of the study conducted in Wuhan, China between April 2020 and May 2020 [8]. Although there are likely a number of potential explanations for these observations, these data need to be put into the context of patient survival [8]. Collectively, the paradoxically decreased treatment delays during the COVID-19 pandemic revealed in our and previous studies [8] suggest successful implementation of efforts to maintain optimal care for neurooncological patients during the COVID-19 pandemic at least at some centers. The observation of larger tumors in our COVID-19 patient cohort and data from Wuhan [8] that indicated significantly larger preoperative tumor sizes during the initial COVID-19 outbreak suggest a potential delay in patients seeking and/or receiving appropriate diagnostic workup for brain tumor symptoms. Ultimately, the almost identical survival curves observed in the two patient groups evaluated in our study strongly implicate no negative impact of the COVID-19 pandemic on overall HGG patient survival.

A valid interpretation of these results warrants a more in-depth analysis of the specific circumstances that determined brain tumor patient treatment at our institution during the first year of the COVID-19 pandemic. As demonstrated previously, the occupancy rate of beds for COVID-19 patients in Austria only reached 18% during the first COVID-19 wave in early 2020 and, thus, unlike in other regions, brain tumor treatment was primarily hindered by preventive measures rather than ICU bed shortages [16, 17]. At our neurosurgical ICU in particular, no COVID-19 patients were treated during the study period and available ICU capacity for was almost identical to before the pandemic. Furthermore, under non-pandemic conditions, typically up to 20% percent of the weekly work time of academic physicians at the Medical University of Vienna is dedicated to scientific and educational activities, which may have ensured availability of crucial personnel capacities during the pandemic so that the first year of the pandemic was manageable without significant decreases in patient numbers despite mandatory quarantine absences and deployment of neurosurgeons to COVID-19 wards. While prioritization of critically ill patients was necessary and neurooncological cases were thus given preference in the elective program and some cancellations of non-oncological cases had to be accepted, the absolute number of surgical procedures performed at our department in 2020 increased by 15% as compared with 2019. This may be due to a higher number of referrals to our tertiary center based on less capacity shortages than at smaller local hospitals. Considering previous findings that demonstrated less changes to care as well es lower mortality rates in high income countries during COVID-19, the availability of these resources may have greatly contributed to the comparatively mild effects of the pandemic observed in this study [14].

Our data strongly indicates that, despite government-implemented restrictive measures aimed at preventing a collapse of the Austrian health care system, adequate treatment of HGG patients at our institution was maintained during the COVID-19 period, without a notable negative impact on patient survival.

### Limitations

There are limitations that should be considered when interpreting the data presented in this study. Firstly, data collection was performed retrospectively. While acquisition of the main variables may still be reliable, it should be considered that patients were, nevertheless, included on the basis of histopathologically confirmed rather than radiologically suspected HGG diagnosis. This is prone to potential selection bias as patients with an unfavorable prognosis may not have been offered surgery due to a COVID-19-related treatment triage. However, we did not identify any cases that were refused treatment at our department during the COVID-19 period. Secondly, the survival examination period was longer in the control group than the COVID-19 group due to a uniform cutoff date for both groups. While this results in a potential bias in the presented survival data, the fact that the 1-year survival rate was even slightly higher during the pandemic suggests that there was no marked increase in mortality. Thirdly, all data discussed in this manuscript are solely based on findings at our department. Therefore, our results cannot be generalized to other hospitals that may have dealt with the pandemic under different hospital-specific circumstances, or had different resource capacities, such as for example smaller local hospitals. Nonetheless, it is crucial to gain better insight on how the COVID-19 pandemic may have impacted patient treatment and outcome, where our data provide a comprehensive estimation of how unprecedented events may affect neurooncological patients in different health care environments.

## Conclusions

This study provides further evidence that the COVID-19 pandemic did not negatively affect the overall survival of HGG patients based on data from our institution in a high income country. While a previous study found preoperative tumor volumes to be significantly higher in HGG treated during COVID-19 [8], the moderately higher tumor volumes in our COVID-19 patient cohort did not reach statistical significance. The reduced time interval between initial referral for surgical treatment to surgery may reflect the focused and efficient allocation of resources to care for critically-ill patient populations, such as patients suffering from HGG.

## Abbreviations

COVID-19: Coronavirus disease 2019
HGG: High-grade glioma
IDH: Isocitrate dehydrogenase
IQR: Interquartile range
MRI: Magnetic resonance imaging
WHO: World Health Organization
